# The Effect of an Extract and Fractions of Date Pits on Some Plasma Constituents, Reproductive Hormones, and Testicular Histology in Male Mice

**DOI:** 10.1155/2022/4102960

**Published:** 2022-10-25

**Authors:** Mohammed Al Za'abi, Badreldin H. Ali, Haytham Ali, Priyadarsini Manoj, Mohammed Ashique, Abderrahim Nemmar, Lucie Cahlíková

**Affiliations:** ^1^Department of Pharmacology and Clinical Pharmacy, College of Medicine and Health Sciences, Sultan Qaboos University, Muscat, Oman; ^2^Department of Animal and Veterinary Sciences, College of Agricultural and Marine Sciences, Sultan Qaboos University, Muscat, Oman; ^3^Department of Physiology, College of Medicine and Health Sciences, UAE University, Al Ain, UAE; ^4^ADINACO Research Group, Faculty of Pharmacy, Charles University, Prague, Czech Republic

## Abstract

Pits of dates (*Phoenix dactylifera* L.) have numerous nutritional benefits that could have wide-ranging applications. This study aimed to examine the effects of administering three extracts from powdered date pits on some basic physiological parameters, plasma constituents, reproductive hormones, and testicular histology in CD1 male mice. Three groups received doses of 100 mg/kg/day of lyophilized extract, a nonpolar fraction, and a polar fraction of date pits by oral gavage for 28 consecutive days. For the control, one group was administered a 1 mL/kg concentration of distilled water. The three different extracts significantly increased the plasma testosterone level but showed no significant effect on estradiol or luteinizing hormone levels, except for estradiol reduction in the polar extract group. The measured physiological or biochemical parameters or testicular histology also demonstrated no significant differences between the control mice and those mice treated with the three extracts, except for reductions in plasma urea in all extracts and in total protein in the nonpolar extract. Therefore, date pit extracts may potentially be used as a safe and effective dietary supplement. However, further investigation is needed.

## 1. Introduction

Dates (*Phoenix dactylifera* L.) have been an ancient staple crop cultivated in the arid regions of the Arabian Peninsula for over 5000 years [1, 2]. The date flesh fruit contributes to people's food security, nourishment, and healthy life improvement in North Africa and the Middle East [3]. Over the centuries, date pits have been provided significant nutritional, medicinal, and economic value to humans and animals. They can be used efficiently as a nontraditional feed in the diets of farm animals such as goats, chickens, and fish [4], enhancing their body weight gain, feed efficiency, and meat palatability [5]. In the food industry, date pits have been utilized experimentally to preserve and enhance the stability during the shelf life of beef burgers and could improve the composition of bioactive compounds (fiber and phenolic content) of such foods [6].

Phytochemically, date pits are a rich source of polyphenols and flavonoids [7]. Pharmacologically, date pits in cells, animals, or humans possess antioxidant [8], anti-inflammatory [9], antidiabetic [10], and antibacterial [11, 12] properties.

Moreover, date palm, date palm pollen, and pit reportedly have therapeutic effects on human infertility [13,14]. A study on the effect of date pit powder on nicotine-induced spermatotoxicty in adult albino mice showed that date pits caused a statistically significant increase in the Johnsen score and seminiferous tubule diameter [15]. Abdallaha et al. [16] also demonstrated that date pit supplementation alleviated reproductive disorders in male diabetic rats. The activation of the testicular endocrine and antioxidant systems by date pits' phytochemicals, such as flavonoids, carotenoids, and anthocyanins, may lead to these effects.

However, the effects of date pit intake on reproductive hormones are varied. For example, Elgasim et al. [17] fed the sheep with date flesh and pits and found that the sheep's body weights and back fat disposition increased; in addition, the extracts of date flesh or pits increased the uterine weight of immature rats and induced uterine contraction *in vitro*. Therefore, Elgasim et al. [17] hypothesized that date flesh and pits contain estrogen. Ali et al. [18] showed that date pit feeding among male rats (7% and 14%) for 4 weeks increased testosterone levels. This result was later confirmed by Orabi and Shawky [19]. In contrast, Aldhaheri et al. [20] reported that date pits had no effect on the testosterone levels of male rats but significantly decreased the plasma estradiol levels of female rats. Currently, we found no published articles regarding the effect of date pits on male reproductive hormones. Therefore, this study aimed to investigate the effects of three preparations of date pit extracts on the reproductive hormones of male mice and to compare the findings with previously obtained data from male rats.

## 2. Materials and Methods

### 2.1. Animals

Male CD1 mice (9–10 weeks old, initially weighing approximately 25 g) were obtained from the Small Animal House of Sultan Qaboos University. They were housed at a room temperature of 22°C ± 2°C and relative humidity of approximately 60%, with a 12 h light-dark cycle (lights on at 6 : 00 A.M.). They were fed with a standard diet and tap water. The Sultan Qaboos University (SQU) Animal Ethics Committee approved this study (SQU/AEC/2019-20/11), and all procedures involving animals and their care conformed to the international laws and policies (EEC Council directives 2010/63/EU, September 22, 2010, and NIH Guide for the Care and Use of Laboratory Animals, NIH Publications, 8th edition, 2011).

### 2.2. Preparation of Date Pit Extracts

According to the method described by Ali et al. [18], one of the authors of the present study (Lucie Cahlíková) prepared the extracts obtained from Omani date (Khalas) pits.

#### 2.2.1. Lyophilized Extract (D1)

These Khalas date pits were coarsely powdered, and 200 g were macerated with distilled water (1000 mL) for 8 h with occasional shaking. The extract was filtered and concentrated to 200 mL. The extract volume was freeze-dried to obtain a lyophilized extract (6.628 g).

#### 2.2.2. Nonpolar Fraction (D2)

Coarsely powdered date pits (500 g) were extracted with hexane by using a Soxhlet extractor. The hexane extract was evaporated under a vacuum to give an oily yellowish-brown residue (35.6778 g).

#### 2.2.3. Polar Fraction (D3)

The residue yield after hexane extraction was dried and macerated with distilled water (4000 mL) for 6 h with occasional shaking. The extract was filtered and lyophilized to give a dark-brown extract (17.780 g).

### 2.3. Chemical Characterization of Date Pit Extracts by GC-MS

GC-MS analysis was performed on an Agilent 890A GC 5975 inert MSD operating in EI mode at 70 eV (Agilent Technologies, Santa Clara, CA, USA). The separation was carried out on a DP-5 MS column (30 m × 0.25 mm × 0.25 *μ*m, Agilent Technologies, Santa Clara, CA, USA). The temperature program was from 100–150°C at 15°C/min, 1 min hold at 180°C, and then 180°C-300°C at 5°C/min, and 35 min hold at 300°C. The injector temperature was 280°C. The flow rate of carrier gas (helium) was 0.8 mL/min. The detection range was m/*z* 35–600, and the detector temperature was 200°C. An injection of 1 *μ*L of solution (1 mg/mL) was introduced in split mode (split ratio 1 : 10). The individual components were identified based on a comparison of their MS with those in the National Institute of Standards and Technology (NIST) library (MD, USA).

### 2.4. Experimental Design

The mice (*n* = 28) were randomly distributed into four equal groups and were treated for 4 weeks as follows:

- Group 1 (control): mice received distilled water (1 mL/kg) until the end of the study.
- Group 2 (lyophilized extract group, D1): mice received lyophilized date pit extract (D1) dissolved in distilled water at a dose of 100 mg/kg/day orally daily for 28 days.
- Group 3 (nonpolar fraction group, D2): mice received a nonpolar fraction of date pit extract (D2) suspended in distilled water at a dose of 100 mg/kg/day orally daily for 28 days.
- Group 4 (polar fraction, D3): mice received a polar fraction of date pit extract (D3) dissolved in distilled water at a dose of 100 mg/kg/day orally daily for 28 days.

On the last treatment day, the mice were placed individually in metabolic cages to facilitate the measurement of feed, water intake, urinary output, and fecal output. Immediately after 24 hours of metabolic cage placement, they were weighed and anesthetized with ketamine (75 mg/kg) combined with xylazine (5 mg/kg) intraperitoneally. Blood samples were then collected from the inferior vena cava in heparinized tubes and centrifuged at 900 g for 15 min at 4°C to separate plasma. The collected plasma was stored frozen at −80°C awaiting biochemical analysis within 10 days. The mice were then sacrificed by anesthesia overdose. Next, the testes were resected, washed with ice-cold saline, blotted with a piece of filter paper, and weighed. A small part of the right testis was fixed in modified Davidson's fluid for histopathological analysis.

### 2.5. Biochemical Analysis

The concentration levels of plasma urea, total protein, albumin, and total cholesterol and the activities of alanine aminotransferase (ALT), aspartate aminotransferase (AST), and alkaline phosphatase (ALP) were measured using an automated biochemical analyzer (Mindray BS-120 Chemistry Analyzer; Shenzhen Mindray Bio-Medical Electronics Co., Shenzhen, China) as previously described [21].

### 2.6. Hormonal Analysis

We measured the plasma concentrations of testosterone (Catalog no. EIA1559) and luteinizing hormone (LH) (Catalog no. EIA1289) by using a DRG Instruments GmbH ELISA kit (Marburg, Germany), and the estradiol concentration (Catalog no. CSB-E05109m) by using the ELISA kits (Cusabio Biotech Co., Ltd., Wuhan, Hubei Province, P.R. China).

### 2.7. Histopathological Analysis

The testis samples were fixed in Davidson's fixative for 24 h and then transferred to 10% neutral buffer formalin. These samples were routinely processed, and 4 *μ*m sections were stained with hematoxylin periodic acid Schiff (HPAS) (ab150680, Abcam, USA), and then examined under an Olympus BX51 microscope attached to an Olympus DP70 camera.

Histopathological grading was performed according to the Cosentino et al., [22] scoring system from I-IV as stated in Table 1.

### 2.8. Statistical Analysis

The data are presented as the mean ± SEM and were analyzed by one-way analysis of variance followed by Bonferroni's multiple comparison test (GraphPad Prism version 5.03, San Diego, CA, USA). A *P* value of less than 0.05 was considered statistically significant.

## 3. Results

### 3.1. Chemical Characterization of Date Pit Extracts by GC-MS

**Figure 1:**
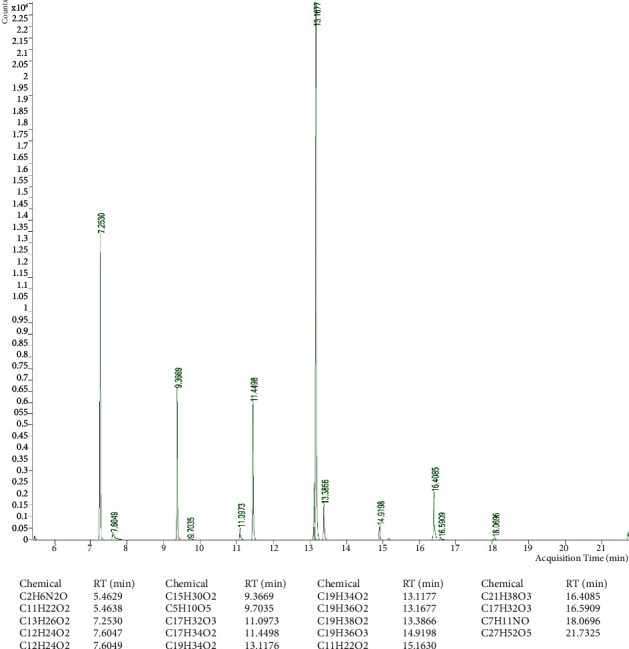
GC-MS chemical characterization of a lyophilized extract of date pits. RT: retention time.

**Figure 2:**
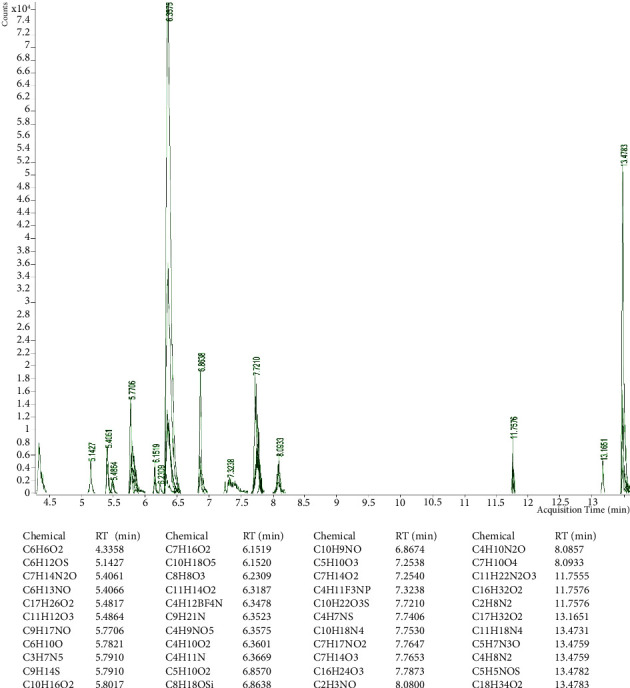
GC-MS chemical characterization of a nonpolar extract of date pits. RT: retention time.

**Figure 3:**
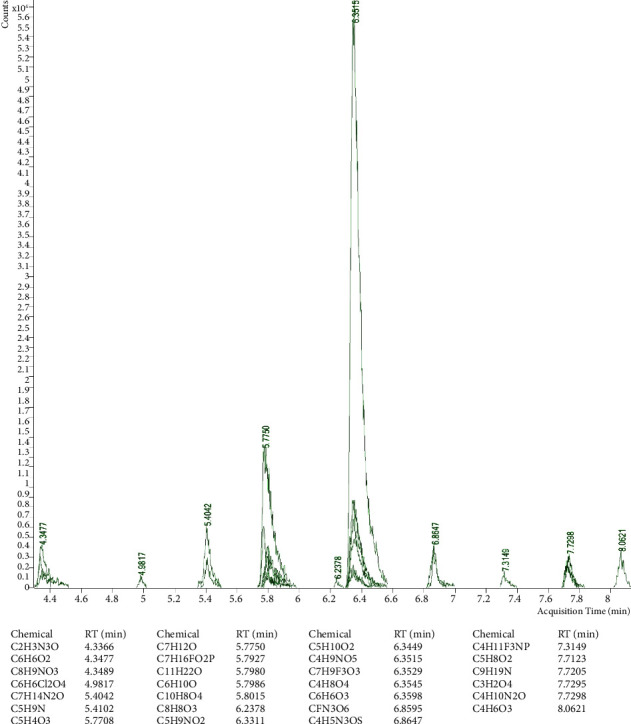
GC-MS chemical characterization of a polar extract of date pits. RT: retention time.

The major components of the lyophilized extract (Figure 1) were: methyl-9-octadecenoate (RT = 13.1677 min), methyldodecanoate (RT = 7.2530 min), methyltetradecanoate (RT = 9.3669 min), methylhexadecanoate (*R* = 11.4498) and methyl-9,12-octadecedienoate (RT = 13.1176 min). The main components of nonpolar fractions (Figure 2) were identified as: 2-hydroxymethyl-2-nitro-1,3-propandiol (RT = 6.3515 min), 4-hydroxy-2-methyl-2-butanone (RT = 6.3449 min) and 5-methyl-3-hex-2-one (RT = 5.775 min). 2-Hydroxymethyl-2-nitro-1,3-propandiol (RT = 6.3575 min), oleic acid (RT = 13.4783 min), and 2-methyl-3-but-2-ol (RT = 6.8638 min) were the major components of the polar extract (Figure 3).

### 3.2. Physiological Data

Table 2 lists the effects of different date pit extracts on some physiological parameters. Compared with the control, all extracts reduced the weight, but only the effects of lyophilized and polar extracts were statistically significant. The effects of the three extracts on total and relative testes weight, water and food intake, and urine and fecal outputs were not significantly different from those of the control, except for the lyophilized extract effect on food intake.

### 3.3. Biochemical Data

Table 3 shows the effect of the three extracts on some plasma parameters. The nonpolar extract significantly reduced the total proteins when compared with the control. All the extracts significantly reduced the urea, but the effects on albumin, ALP, AST, ALT, and total cholesterol were not significant.

### 3.4. Hormones

**Figure 4:**
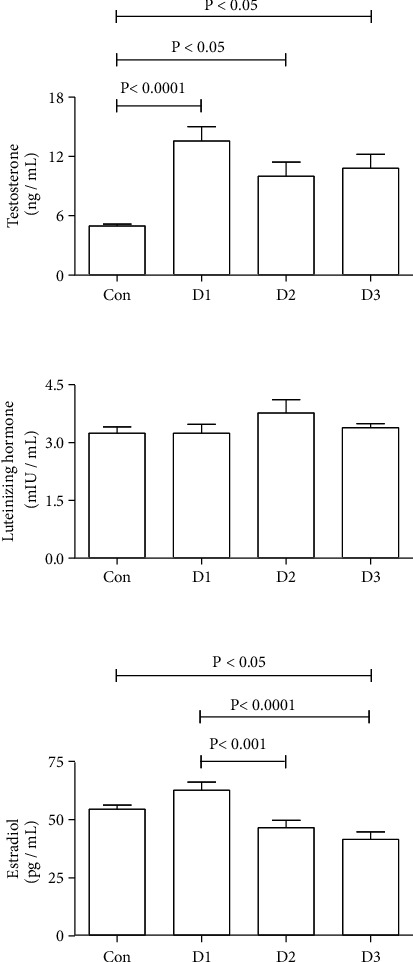
Effect of date pit extracts on testosterone, luteinizing hormone (LH), and estradiol levels in mice. The column and vertical bar represent the mean ± SEM (*n* = 6). Extracts (100 mg/kg): D1: lyophilized; D2: nonpolar fraction; and D3: polar fraction. These extracts were given to mice throughout the experiment by oral gavage. On treatment day 28, the mice were sacrificed for plasma collection. Con: control. Differences between the groups were assessed by one-way analysis of variance (ANOVA) followed by Bonferroni's multiple comparison test, where *P* < 0.05.

Figure 4 depicts the effects of the three extracts on testosterone, LH, and estradiol concentrations. All extracts significantly elevated the testosterone concentration levels when compared with the control. In comparison with the control, lyophilized, or nonpolar extract, the polar extract significantly decreased the estradiol concentration levels. Meanwhile, none of the extracts significantly changed the LH concentration levels.

### 3.5. Histology

**Figure 5:**
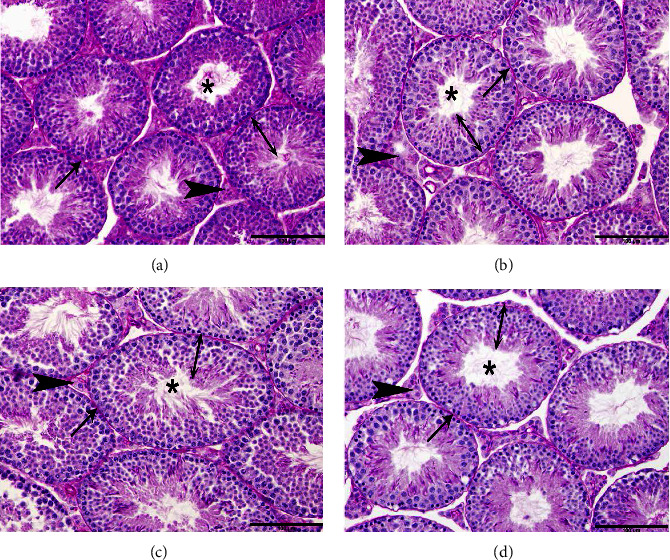
Photomicrograph of mice's testis (Bar = 100 *μ*m; Stain: HPAS); (A–D) groups 1–4, showing normal histological structures of the seminiferous tubule, with a complete spermatogenesis process (double arrow), mature Sertoli cells (arrows), intact Leydig cells (arrowheads), and an appropriate percentage of mature spermatozoa (asterisks) (corresponds to grade I Cosentino grading classifications). (a) control; (b) lyophilized extract; (c) nonpolar fraction extract; and (d) polar fraction extract.

Figure 5 shows the effect of the extracts on testicular histology. In the examined testicular sections, the histological structures of the seminiferous tubule were normal in all groups, with a complete spermatogenesis process, mature Sertoli cells, intact Leydig cells, and an appropriate percentage of mature spermatozoa (Figures 5(a)–5(d)). The scores of all examined testicular tissues from all mice of all groups were grade I.

## 4. Discussion

Date pits, which account for 10%–15% of the weight of a date palm depending on the variety, have several nutritional benefits that may have extensive potential applications [5, 23]. This study specifically used Khalas date pits to examine the date pits' effects on numerous physiological and biochemical parameters, as well as the concentrations of three reproductive hormones, in male mice. The proximate analysis and dietary fiber composition of this type of date pit and date palm were described previously [24].

In this study, the three different extracts of date pits significantly increased the plasma testosterone levels of treated mice, but only the polar extract significantly reduced the estradiol levels. None of the extracts had a statistically significant impact on LH levels. These results are consistent with Ali et al.'s studies (1999), wherein the inclusion of 7%–14% of powdered date pits in the diet of male rats increased their testosterone levels while having no effect on their estradiol or LH levels. The observed increase in testosterone levels, however, was not manifested in any overt androgenic metabolic-related actions. Furthermore, the experimental mice's body weight and any gross or histological changes in the testicles did not increase. Almost all of the other physiological and biochemical parameters measured for all extracts were not statistically different from those measured for the controls.

The three different extracts from these date pits did not significantly impact the physiological parameters measured in the study. Additionally, these extracts had no effect on the biochemical parameters, except for the observed reduction in urea by all extracts and in the total proteins by the nonpolar fraction. To our knowledge, the effect of date pits on urea, cholesterol, or proteins remains uninvestigated. Nonetheless, date palm fruit is beneficial in lowering low-density lipoprotein cholesterol levels and counteracting elevated urea in rats exposed to trichloroacetic acid [25, 26].

The presence of several chemical compounds within the date pits is responsible for these effects. In addition to minerals such as zinc, cadmium, calcium, and potassium, compounds similar to saturated fatty acids such as palmitic acid and stearic acid, as well as unsaturated fatty acids, such as oleic acid and linoleic acid, are found [27,28]. Date pits also contain antioxidants such as carotenoids, anthocyanins, and glycosidic flavonoids derived from the flavone, flavonol, and flavoxanthine classes, considering their high percentage of antioxidant activity [27].

These chemicals may improve male reproductive parameters such as hormonal levels and seminal vesicle parameters, as well as sperm motility, count, and viability, and facilitate the binding to estrogen receptors. Indeed, data products exert a protective role against cadmium-induced [29] or streptozotocin-induced [30] infertile male rats. Additionally, date pollen administration to humans increased their sperm count and motility, as well as their LH and testosterone levels [31].

## 5. Conclusions

The three different extracts significantly increased the plasma testosterone level, with little or no significant effect on estradiol or LH levels. However, the short- or long-term effects associated with testosterone level increases in other organs warrant further studies.

## Figures and Tables

**Table 1:** Histological grading system of testicular damage according to the Cosentino^*∗*^ classification.

Grade I	Normal testicular architecture with an orderly arrangement of germinal cells.
Grade II	Less orderly, noncohesive germinal cells and closely packed seminiferous tubules.
Grade III	Disordered sloughed germinal cells with shrunken pyknotic nuclei and less distinct seminiferous tubule borders.
Grade IV	Seminiferous tubules are closely packed with coagulative necrosis of the germinal cells.

^*∗*^ [22]. Histological changes occur in the contralateral testes of prepubertal rats subjected to various durations of unilateral spermatic cord torsion. J Urol. 133, 906-11.

**Table 2:** Effects of three different date pit extracts on some physiological parameters in mice.

Parameters/Treatment	Control	Lyophilized extract	Nonpolar fraction extract	Polar fraction extract
Body weight change (%)	1.48 ± 0.47	−3.72 ± 0.84^a^	−1.68 ± 1.41	−3.25 ± 0.50^a^
Total testes weight (g)	0.22 ± 0.008	0.23 ± 0.007	0.23 ± 0.02	0.22 ± 0.01
Relative testes weight (%)	0.58 ± 0.02	0.64 ± 0.03	0.62 ± 0.04	0.59 ± 0.03
Water intake (mL)	4.99 ± 0.03	6.14 ± 0.70	6.4 ± 0.69	5.2 ± 0.16
Urine output (mL)	1.56 ± 0.19	1.96 ± 0.25	1.09 ± 0.04	1.34 ± 0.21
Food intake (g)	3.04 ± 0.57	1.47 ± 0.19^a^	1.91 ± 0.23	2.69 ± 0.23
Feces output (g)	1.04 ± 0.22	1.21 ± 0.15	1.06 ± 0.04	0.8 ± 0.15

The values in the table are the means ± SEM (*n* = 7). Extracts (100 mg/kg) were given to mice throughout the experiment by oral gavage. On the 28th day of treatment, the mice were sacrificed to collect plasma. Differences between the groups were assessed by one-way analysis of variance (ANOVA) followed by Bonferroni's multiple comparison test, where *P* < 0.05. ^a^denotes the significance (*P*=0.0015) of the control group *vs*. different groups. ^b^denotes the significance (*P*=0.0129) of the control group vs. different groups.

**Table 3:** Effects of three different date pit extracts on some plasma parameters in mice.

Parameters/Treatment	Control	Lyophilized extract	Nonpolar fraction extract	Polar fraction extract
Total proteins (g/dL)	5.25 ± 0.36	4.37 ± 0.14	4.18 ± 0.23^a^	4.79 ± 0.13
Albumin (g/dL)	1.98 ± 0.12	1.78 ± 0.18	1.46 ± 0.19	1.87 ± 0.08
Alkaline phosphatase (U/L)	18.9 ± 0.95	14.74 ± 1.88	17.09 ± 2.81	20.87 ± 2.43
Aspartate aminotransferase (U/L)	42.93 ± 3.25	53.44 ± 7.37	49.13 ± 6.38	53.40 ± 7.32
Alanine aminotransferase (U/L)	18.49 ± 0.81	16.51 ± 1.94	16.34 ± 2.67	20.44 ± 2.16
Urea (mmol/L)	4.81 ± 0.45	2.75 ± 0.28^a^	2.34 ± 0.41^a^	2.26 ± 0.41^a^
Total cholesterol (mmol/L)	2.32 ± 0.21	2.64 ± 0.30	1.59 ± 0.22	2.10 ± 0.12

The values in the table are the means ± SEM (*n* = 7). Extracts (100 mg/kg) were given to mice throughout the experiment by oral gavage. On the 28th day of treatment, the mice were sacrificed to collect plasma. Differences between the groups were assessed by one-way analysis of variance (ANOVA) followed by Bonferroni's multiple comparison test, where *P* < 0.05. ^a^denotes the significance (*P*=0.0015) of the control group *vs*. different groups. ^b^denotes the significance (*P*=0.0129) of the control group vs. different groups.

## Data Availability

Data are available upon request to the corresponding author.
